# Effects of emamectin benzoate on pharmacokinetic profiles, bioavailability, and serum biochemical indices in crucian carp (*Carassius carassius*) following oral administration with multiple dosage levels

**DOI:** 10.3389/fvets.2023.1127788

**Published:** 2023-03-06

**Authors:** RuYu Sun, YongTao Liu, XiaoHui Ai, XiangXuan Du, XiaoYi Zhang

**Affiliations:** ^1^Yangtze River Fisheries Research Institute, Chinese Academy of Fishery Sciences, Wuhan, China; ^2^College of Fisheries and Life Science, Shanghai Ocean University, Shanghai, China; ^3^Hubei Province Engineering and Technology Research Center for Aquatic Product Quality and Safety, Wuhan, China; ^4^Key Laboratory of Control of Quality and Safety for Aquatic Products, Ministry of Agriculture and Rural Affairs, Beijing, China; ^5^School of Food Science and Engineering, Bohai University, Jinzhou, Liaoning, China

**Keywords:** emamectin benzoate, pharmacokinetics, bioavailability, serum biochemical indices, crucian carp

## Abstract

The aim of this investigation was to explore the effect of three different dose levels of emamectin benzoate (EMB) (50, 200, and 500 μg/kg bw) on pharmacokinetic characterizations, tissue distribution patterns, absolute bioavailability, and serum biochemical indices in crucian carp following oral administration at 22 ± 2°C, respectively. We further calculated the relevant parameters by detecting the concentration of EMB in the crucian carp by the ultra-HPLC detection method. The results showed that *C*_*max*_, *AUC*, and *T*_1/2*z*_ after oral administration showed a dose-dependent increase in plasma. Secondly, EMB has a long mean flow time (*MRT*) (51.88, 67.04, and 78.00 h, respectively). In conclusion, the elimination of the drug is slow, and the higher dose used, the slower elimination is. The distribution in various tissues of crucian carp was analyzed. The order of EMB levels in tissues of crucian carp was liver > gill > kidney > muscle plus skin > plasma. As for pharmacokinetic parameters in tissues, *C*_*max*_, *AUC*, and *T*_1/2*z*_ in tissues had a similar pattern as that in plasma. The absolute oral bioavailability of EMB (F%) in crucian carp was calculated to be approximately 52.70%. The serum biochemical indices including ALT and AST in experimental groups exhibited significant differences (*P* < 0.05) compared with the control group while ALB, ALP, TP, T-CHO, BUN, GRE, and GLU were not significantly different to the control group (*P* > 0.05). Briefly, EMB has the characteristic of quick absorption and slow elimination in crucian carp with a high bioavailability by PO route in crucian carp.

## 1. Introduction

Crucian carp is one of the most economically important freshwater cultured fish in China. According to the China fishery statistical yearbook in 2021, the production of crucian carp accounted for 8.90% of the total freshwater aquaculture fish production in 2020 (1). However, significant economic loss was frequently caused by parasitic diseases in crucian carp culture (2–4). Emamectin benzoate, a macrolide medicine, exhibited a good effect for controlling infestations both in marine and freshwater-reared fish, and has been approved by the United States Food and Drug Administration (USFDA) and European Medicines Agency (EMA). However, no such regulations are prevailing in most developing countries, including China. In addition, EMB has been given Marketing Authorization by the U.K. Veterinary Medicines Directorate (VMD), and its use in Scotland is increasing as more consents to discharge are issued by the Scottish Environmental Protection Agency (SEPA). EMB is the active ingredient in the sea lice treatment formulation Slice^®^ (Schering-Plough Animal Health), and is administered to infected salmon as an in-feed additive at a dose rate of 50 μg/kg body weight per day, over a 7-day period (5). Furthermore, EMB has a higher safety profile than other similar drugs such as avermectin, ivermectin, doramectin, and so on. EMB will neither bioconcentrate in individual aquatic organisms nor biomagnify in the food chain (6).

Many literatures demonstrated the efficacy of EMB against *Argulus* in zebra fish (*Brachydanio rerio var*) (7), common carp (*Cyprinus flammans*) (8), koi carp (*Cyprinus carpio*) and goldfish (*Carassius auratus*) (9), *endoparasitic monogenean in freshwater-reared largemouth bass* (*Micropterus salmoides*) (10), ectoparasites in seabass (*Lates calcarifer*) (11, 12), and sea lice in salmon (*salmon salar*) (13, 14). The mechanism of EMB against fish parasites is to bind to GABA receptors and cause an increase in membrane chloride permeability, resulting in the paralyzation of parasites that then become ineffective (15–18).

Although EMB has devoted extensive attention owing to their important roles against fish parasites, few investigations on pharmacokinetics of EMB have been reported in aquatic animals such as rainbow trout, salmon, cod, Nile tilapia, and so on (14, 19–21). Information on the disposition of EMB and change of serum biochemical indices after treatment with EMB in crucian carp are even limited, while few studies concentrated on the serum profiles (22, 23). Moreover, only one study has evaluated the absolute oral bioavailability (F%) after EMB treatment (24).

Clearly, there is a need for additional information on the kinetic profile of EMB in aquaculture to further improve treatment efficacy. Additionally, adequate information on the kinetics of the drug is also necessary to help minimize environmental impacts and ensure that treated fish will fit for human consumption. Previous doses used in the literature were 50, 125, 250, and 500 μg/kg bw (25–27). Therefore, the objective of this study is to investigate the pharmacokinetics at the dosage of 50, 200, and 500 μg/kg bw and to calculate related profile parameters of EMB in tissues. Afterward, the bioavailability was calculated at the level of 50 μg/kg bw, and ultimately the serum biochemical indices were detected at three dosage levels. The results will provide useful information to optimize the dosing regimen of EMB in crucian carp.

## 2. Materials and methods

### 2.1. Chemicals and reagents

EMB standard (purity ≥ 97%, CAS number: 155569-91-8) was purchased from Dr. Ehrenstorfer (Augsburg, Germany). SLICE^®^ (EMB; 0.2% aquaculture premix) was supplied by Merck Animal Health (New Jersey, American). HPLC grade acetonitrile, ethyl acetate, and n-hexane used for analysis were all obtained from J. T. Baker (Deventer, Holland). LC-MS grade water was bought from Merck (Darmstadt, Germany). Dimethyl sulfoxide (DMSO) was attained from Sigma-Aldrich (Shanghai, China). Ammonium hydroxide (AR, 25–28%) was ordered from Macklin (Shanghai, China). N-methyl imidazole was purchased from Hwrk Chem (Beijing, China). Magnesium sulfate anhydrous, sodium chloride, heparin sodium, and trifluoroacetic anhydride were purchased from Sinopharm Chemical Reagent Company (Shanghai, China). The centrifugal tubes and 0.22 μm nylon syringe filters were acquired from Shanghai CNW Technologies (Shanghai, China).

Standard stock solution was prepared by dissolving 10 mg of EMB standard in acetonitrile, and then diluted to a final concentration of 100 mg/L. Then, working standard solutions were prepared by diluting the standard stock solution with acetonitrile. All standard solutions were contained in screw thread amber glass bottles and stored at −20°C.

For extractant, 0.2% (*v/v*) ammonium acetonitrile solution was made by the addition of 0.2 mL ammonium hydroxide in 100 mL acetonitrile and mixed well. 0.1% DMSO (*v/v*) solution was made by the addition of 0.1 mL DMSO in 100 mL distilled water. Heparin sodium water solution (1%, *w/v*) was prepared by dissolving 0.1 g of heparin sodium in 10 mL of distilled water.

### 2.2. Experimental animals

The experimental protocols and care of animals were approved by the Fish Ethics Committee of Yangtze River Fisheries Research Institute, Chinese Academy of Fishery Sciences, Wuhan, China (ID 2021-Liu Yongtao-03).

Healthy crucian carp (mean body weight 180 ± 20 g, mixed genders) used in our study were supplied by the aquaculture base of the Yangtze River Fisheries Research Institute, Chinese Academy of Fishery Sciences (Wuhan, China). Before starting the experiment, the fish were acclimated for 1 week. Then, they were raised in tanks supplied with dechlorinated tap water (pH 7.5–8.0). The fish were supplied with oxygen by an inflation pump to ensure the dissolved oxygen levels were kept at 6.0-8.0 mg/L in the water. The nitrite and nitrates were detected every 3 days to keep them at 0.1–0.2 mg/L. Then, the un-ionized ammonia values were kept at < 0.02 mg/L. Otherwise, the water temperature was maintained at 22 ± 2°C by means of an aquarium heater.

### 2.3. Administration

A total of 375 fish were placed in fiberglass tanks (10 fish in each tank) supplied with 300 L dechlorinated tap water. Fish were divided into five groups: the first to third groups for oral administration (PO) study, the fourth group served as the intravenous administration (IV) group, and the fifth group for the vehicle control group. The fish were medicated with a drug-free normal feed for 7 days, then starved for another day before the beginning of the drug administration.

The EMB, as Slice^®^ (Merck Animal Health, New Jersey, American), is administered at 50 μg/kg biomass/day for 7 consecutive days as an in-feed therapeutant against fish ectoparasites. The drug used in the study contains 2 mg of EMB in each gram. In brief, the EMB solutions for oral administration were prepared by dissolving with 0.1% DMSO and adjusting to the final concentrations of 50, 200, and 500 μg/kg bw.

The fish were single orally administered EMB solution with 50, 200, and 500 μg/kg bw by a preloaded syringe down the esophagus (28). As for IV treatment, a single 50 μg/kg bw dose of EMB was injected into the caudal vein (29, 30).

### 2.4. Sample collection

After treatment, each fish was observed, if the EMB solution was regurgitated, the fish was excluded and replaced. The fish were first anesthetized with tricaine methanesulfonate (MS-222, 200 mg/L) (30), and then approximately 2 mL of blood from each of 5 fish at each time point was drawn from the caudal vein using 2 mL heparinized syringe with a 26 G × 1 inch needle on 0.5, 1, 2, 4, 6, 8, 10, 12, 24, 48, 72, 96, 120, 144, and 168 h. After collecting blood, the fish were immediately euthanized by immersion in an overdose of 250 mg/L MS-222 solution (31). Then, muscle plus skin, liver, kidney, and gill were collected at each time point described above. Plasma was isolated from the blood by centrifugation at 3,000 r/min for 5 min and stored at −80°C until analysis. Placed blood stored at 4°C and kept overnight, and then, take the upper layer of serum. Tissues were homogenized by a homogenizer and kept at −80°C until determination.

### 2.5. Sample preparation protocols

The preparation procedure of samples was based on our previous method with some modifications (32).

#### 2.5.1. Sample extraction protocols

In brief, the sample was thawed at room temperature, and 1 mL plasma was transferred into a 10 mL centrifuge tube. Then 1 mL 50 mmol phosphate buffer (PBS) was added into the sample, followed by vortexing for 30 s. Subsequently, 4 mL ethyl acetate was pipetted to the sample, then vortexed for 1 min. Next, the sample was centrifuged at 5,000 r/min for 5 min, and the supernatant was decanted into a new 10 mL tube. The ethyl acetate extract procedures described above were repeated and the extracts were combined together.

After defrosting 1 g tissue sample was weighed into a 10 mL centrifuge tube. 4 mL 0.2% ammonium acetonitrile solution was added, then vortexed for 30 s and ultrasonically extracted for 2 min. Next, 0.5 g anhydrous magnesium sulfate was weighed into them, followed by shaking vigorously for 30 s. After centrifugation for 5 min at 5,000 rpm, the supernatant was transferred into a new 10 mL centrifuge tube. The residues were re-extracted with 3 mL 0.2% ammonium acetonitrile solution. After that, the obtained upper layer was combined, and then 2 mL n-hexane saturated with acetonitrile was added into the tube, followed by vortexing for 30 s. After centrifugation at 5,000 rpm for 5 min, the n-hexane layer was removed.

#### 2.5.2. Derivatization reaction

The tubes filled with extract solution were condensed to dryness by a gentle nitrogen stream at 40°C (plasma) or 50°C (liver, kidney, gill, and muscle plus skin). Afterward, 100 μL of derivatization reagent A [N-methyl imidazole with acetonitrile (1:1, *v/v*)] and 150 μL of derivatization reagent B [trifluoroacetic anhydride with acetonitrile (1:2, *v/v*)] were added into the tubes. After vortexing evenly for 30 s, the derivatization reaction lasted for 20 min at room temperature. Subsequently, the derivative was reconstituted by 1 mL acetonitrile. Finally, the obtained solution was filtered through 0.22 μm nylon syringe filter for ultra-high performance liquid chromatography-fluorescence (UHPLC/FLD) analysis.

### 2.6. Instrument analysis

All samples were analyzed by an Agilent UPLC (Milford, MA, USA) equipped with quart solvent manager with a quart solvent pump, a sampler manager with an autosampler, and a fluorescence detector (FLD). An Infinity Lab Poroshell 120 EC-C18 column (2.7 μm, 100 × 4.6 mm) (Agilent, USA) was used for separation. The column temperature was set at 40°C, and the injection volume was 20 μL. The detector was operated at an excitation wavelength of 365 nm and an emission wavelength of 465 nm, respectively. The mobile phase consisted of water and acetonitrile and was applied at a flow rate of 1 mL/min. The gradient elution procedure is shown in Table 1.

**Table 1:** Gradient elution program for EMB separation.

**Time (min)**	**Water (%)**	**Acetonitrile (%)**
0	6	94
6	6	94
6.1	1	99
8	1	99
8.1	6	94
10	6	94

### 2.7. Method validation

According to (33) guidelines for the chromatographic method, recovery rate was determined by comparing the measured concentration of blank sample spiked before and after preparation. Samples were treated as description of 2.4, and each concentration was assayed in six replicates. Intra-day and inter-day precision were evaluated by calculating the relative error (RE) and relative standard deviation (RSD).

Standard calibration curve for EMB was constructed by plotting EMB peak areas vs. seven EMB concentrations (5, 10, 50, 100, 200, 500, and 1,000 μg/L) in acetonitrile.

Recovery was determined by analyzing samples spiked at four levels (5, 10, 50, and 200 μg/L or μg/kg for EMB in plasma and various tissues) in five replicates.

As for intra-day precision of the assay method was determined in a single day, for inter-day precision, five replicates of four different concentration levels were evaluated within 3 days.

### 2.8. Pharmacokinetic profiles and tissue distribution patterns

The pharmacokinetic parameters were calculated by the Phoenix WinNolin 8.1 Software (Certara Corporation, USA), and the concentration-time profiles of EMB were plotted by Prism 8.0 Software (GraphPad Software, USA). The following parameters were estimated: λ*z*: (elimination rate constant), *T*_1/2*z*_: (terminal elimination half-life = 0.693/*Zeta), T*_*max*_: (time to attained maximal concentration), *C*_*max*_: (observed maximal concentration), *AUC*: (area under the concentration-time curve), *MRT*_*last*_: (mean flow time of drug in body from zero to last time point) (34).

### 2.9. Bioavailability

The absolute bioavailability was calculated by comparing the area under the curve (*AUC*) of EMB after PO and IV following the below equation:

$$\begin{array}{l} F\;\left(\%\right)=\frac{\;{AUC\;}_{oral\;administration}\times Dose_{\;intravenous\;administration}}{{AUC\;}_{intravenous\;administration}\times Dose_{\;oral\;administration}}\times 100 \end{array}$$

Dose _oraladministration_ and dose _intravenousadministration_ correspond to the actual doses by oral and intravenous administration, respectively (35).

### 2.10. Serum biochemical indices analysis

Serum contents of glucose (GLU), total cholesterol (T-CHO), blood urea nitrogen (BUN), albumin(ALB), total protein (TP), alkaline phosphatase (ALP), and creatinine (CRE) as well as the activities of aspartate aminotransferase (AST) and alanine aminotransferase (ALT) were assayed by a Chemix-800 automatic biochemical analyzer (Japan Sysmex Corporation, Japan) (36, 37). Significant differences were analyzed by SPSS Statistics 25.0 Software (IBM, USA).

## 3. Results

### 3.1. Method validation

The relevant data of recoveries, intra-day and inter-day precisions in plasma and tissues of crucian carp are listed in Table 2.

**Table 2:** Recovery rates of EMB in plasma and various tissues of crucian carp (*n* = 5).

**Tissues**	**Spiked level (μg/kg)**	**Recovery (%)**	**Precision (CV, %)**	
			**Intra-day**	**Inter-day**
Muscle plus skin	5	88.35	3.53	8.74
	10	89.87	3.44	7.03
	50	88.36	5.33	7.32
	100	86.95	4.12	7.19
Plasma	5	85.74	5.36	8.89
	10	94.79	4.01	8.54
	50	90.26	8.73	11.82
	100	96.11	1.88	8.17
Liver	5	94.74	2.53	7.23
	10	108.48	2.49	6.42
	50	87.96	4.27	3.63
	100	85.99	3.61	1.30
Kidney	5	95.79	2.73	6.23
	10	104.42	3.35	5.07
	50	89.09	2.42	3.33
	100	96.58	1.23	2.85
Gill	5	104.39	2.13	4.34
	10	117.63	3.41	11.24
	50	95.55	2.61	6.79
	100	94.66	1.84	5.87

EMB concentrations and corresponding peak areas in the range of 5–1,000 μg/L showed good linearity with R^2^ ≥ 0.999. The recovery rates varied from 85.74 to 117.63%. The intra-day and inter-day precisions (CV) ranged between 1.23 to 11.82%.

**Figure 1:**
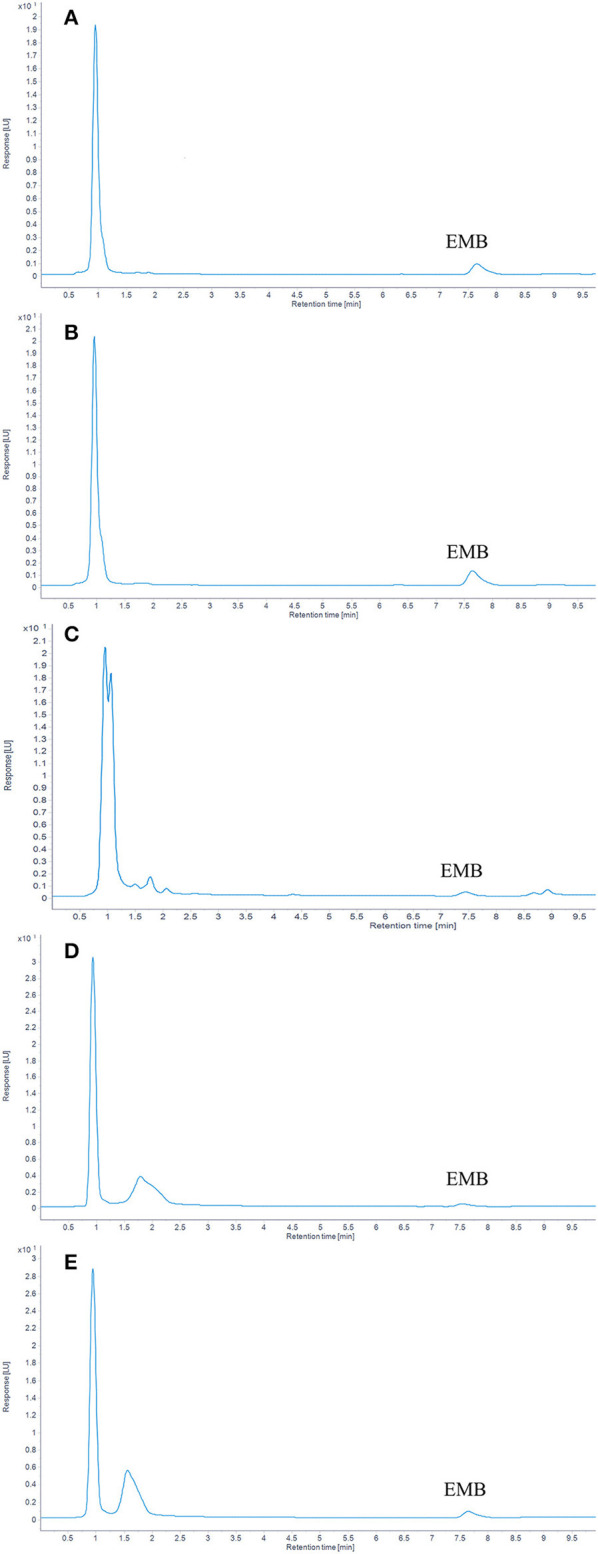
The chromatograms of EMB in tissues. **(A)** Plasma; **(B)** Muscle; **(C)** Liver; **(D)** kidney; **(E)** Gill.

In plasma, the limit of detection (LOD) for EMB was 1 μg/kg (S/N > 3), and the limit of quantitation (LOQ) was 5 μg/kg (S/N > 10). In muscle plus skin, liver, kidney, and gill, the value of LOD, defined by (S/N > 3), was 2 μg/kg and the value of LOQ, defined by (S/N > 10), was 5 μg/kg. The chromatograms of EMB in tissues and plasma are listed in Figure 1.

Consequently, this validated method was reliable for the determination of EMB in plasma and various tissues of crucian carp.

### 3.2. Pharmacokinetic profiles and tissue distribution patterns

**Figure 2:**
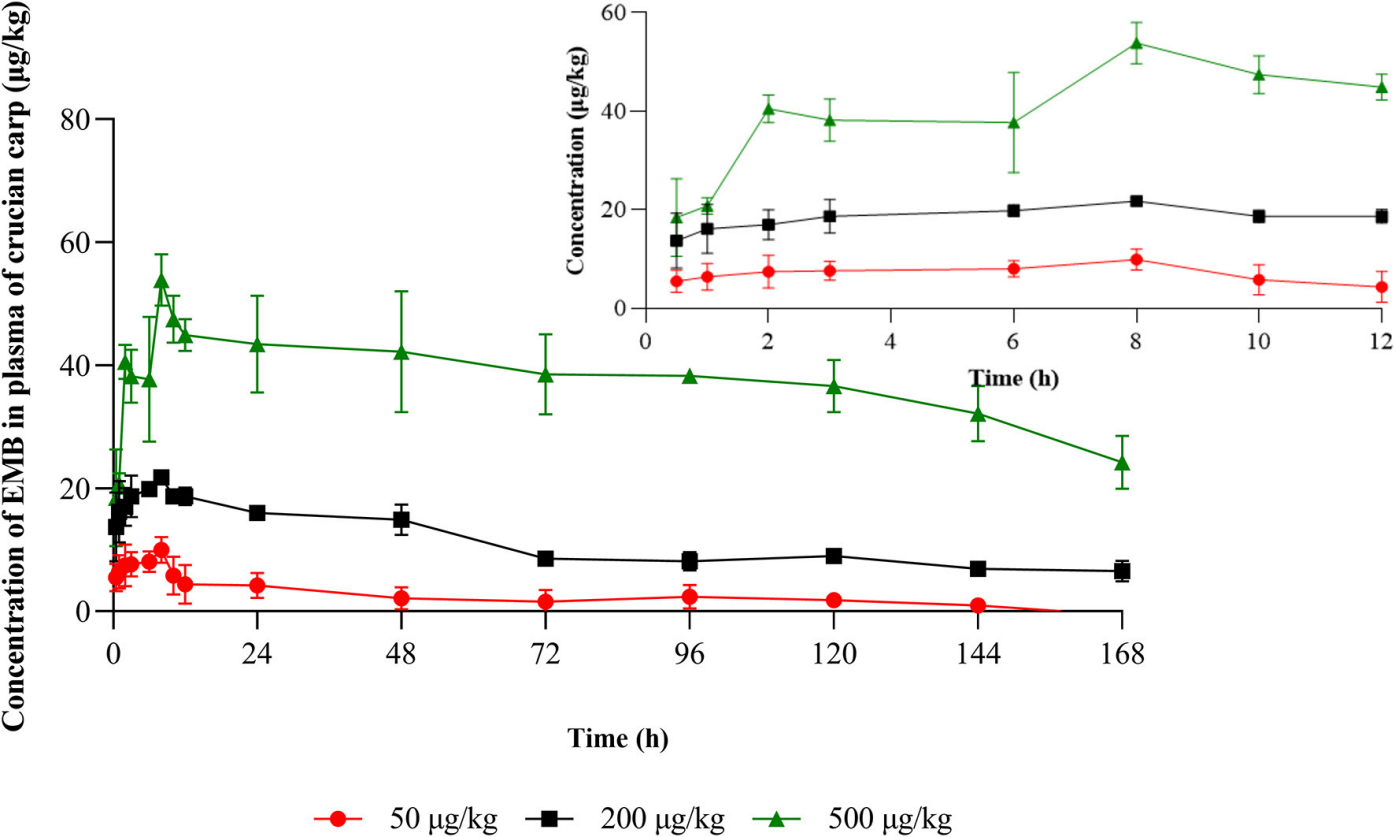
Concentration-time profiles of EMB in plasma of crucian carp after single PO administration of 50, 200, and 500 μg/kg bw at 22 ± 2°C (*n* = 5), respectively.

Plasma concentration-time profiles of the target compound in crucian carp following PO treatment with 50, 200, and 500 μg/kg bw were presented in Figure 2 and the plasma concentration-time profile with IV treatment with 50 μg/kg bw was shown in **Figure 4**. And the relative pharmacokinetic parameters of EMB in crucian carp plasma were documented in Table 3.

**Table 3:** Important pharmacokinetic parameters in plasma of crucian carp following administration with different doses of EMB.

**Parameters**	**Unit**	**Administration**			
		**PO (**μ**g/kg bw)**		**IV (**μ**g/kg bw)**	
		**50**	**200**	**500**	**50**
*λz*	1/h	0.019	0.007	0.008	0.038
*T_1/2*z*_*	h	14.99	39.03	64.87	18.04
*T_*max*_*	h	8.0	8.0	8.0	–
*C_*max*_*	μg/L	9.96	21.77	53.86	–
*AUC_0−168_*	h·μg/L	382.01	1,833.94	6,310.65	724.90
*MRT_*last*_*	h	51.88	67.04	78.00	14.71

λ*z*, elimination rate constant; *T*_1/2*z*_, terminal elimination half-life; *T*_*max*_, time to attained maximal concentration; *C*_*max*_, observed maximal concentration; *AUC*_0−168_, area under the concentration-time curve from 0 to 168 h; *MRTlast*, mean flow time of drug in body from zero to 168 h; PO, oral administration; IV, intravenous administration.

After PO treatment with 50, 200, and 500 μg/kg bw, in plasma, values of *C*_*max*_ for EMB were 9.96, 21.77, and 53.86 μg/L, respectively, and the corresponding *T*_*max*_ were all 8 h. Values of λ*z* were calculated to be 0.019, 0.007, and 0.008 1/h, and *T*_1/2*z*_ were found to be 14.99, 39.03, and 64.87 h, meanwhile, *AUC*_0−168_ were estimated to be 382.01, 1,833.94, and 6,310.65 h·μg /L while *MRT*_*last*_ were 51.88, 67.04, 78.00 h, respectively. For the IV route, pharmacokinetic parameters of EMB in crucian carp plasma were determined. The value of λ*z* for EMB was calculated to be 0.038 1/h, meanwhile, *T*_1/2*z*_ was found to be 18.04 h. The *MRT*_*last*_ was 14.71 h while the value of AUC_0−168_ was estimated to be 724.90 h·μg/L.

**Figure 3:**
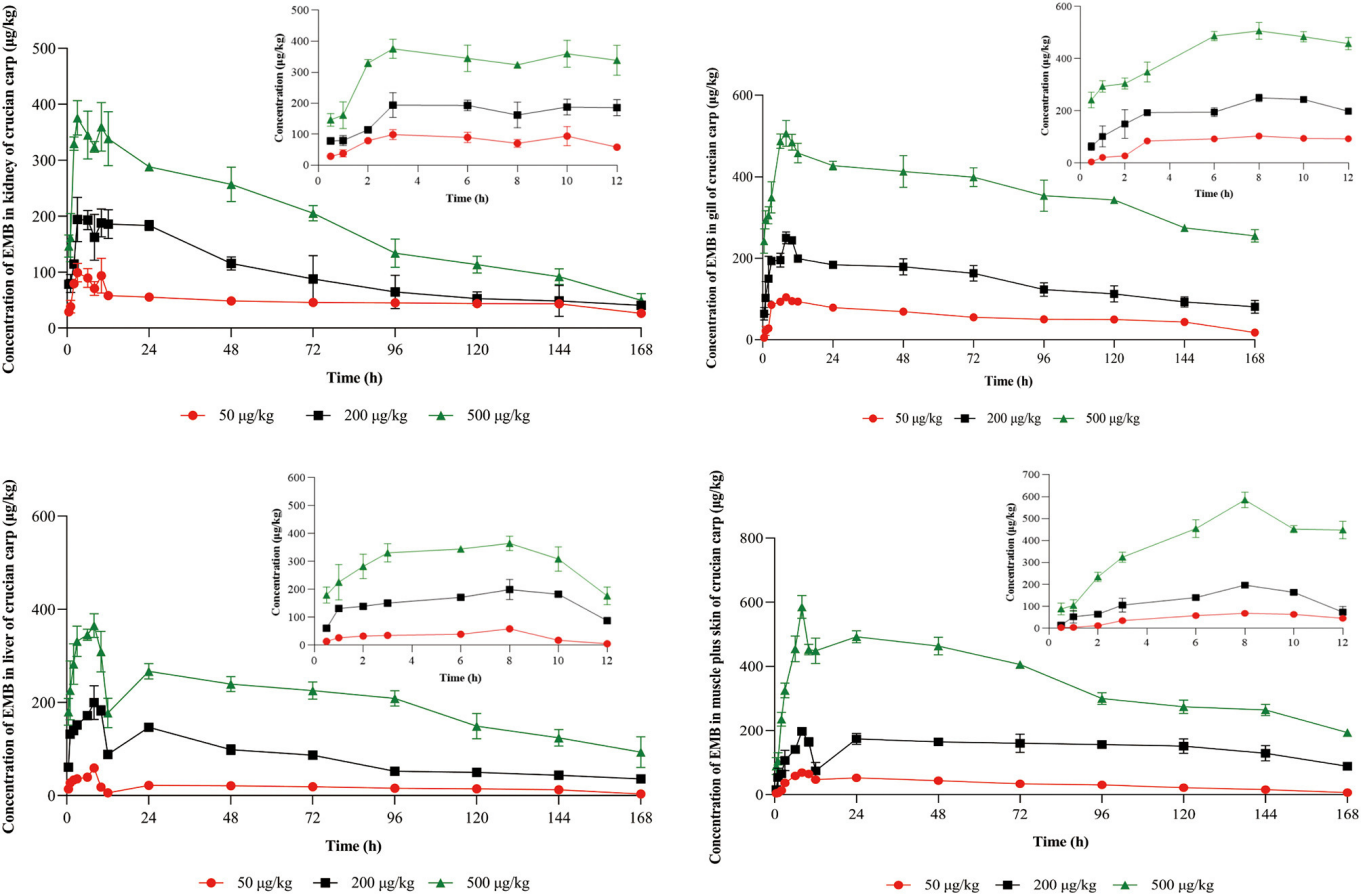
Concentration-time profiles of EMB in tissues of crucian carp after single PO administration of 50, 200, and 500 μg/kg bw at 22 ± 2°C (*n* = 5), respectively.

**Figure 4:**
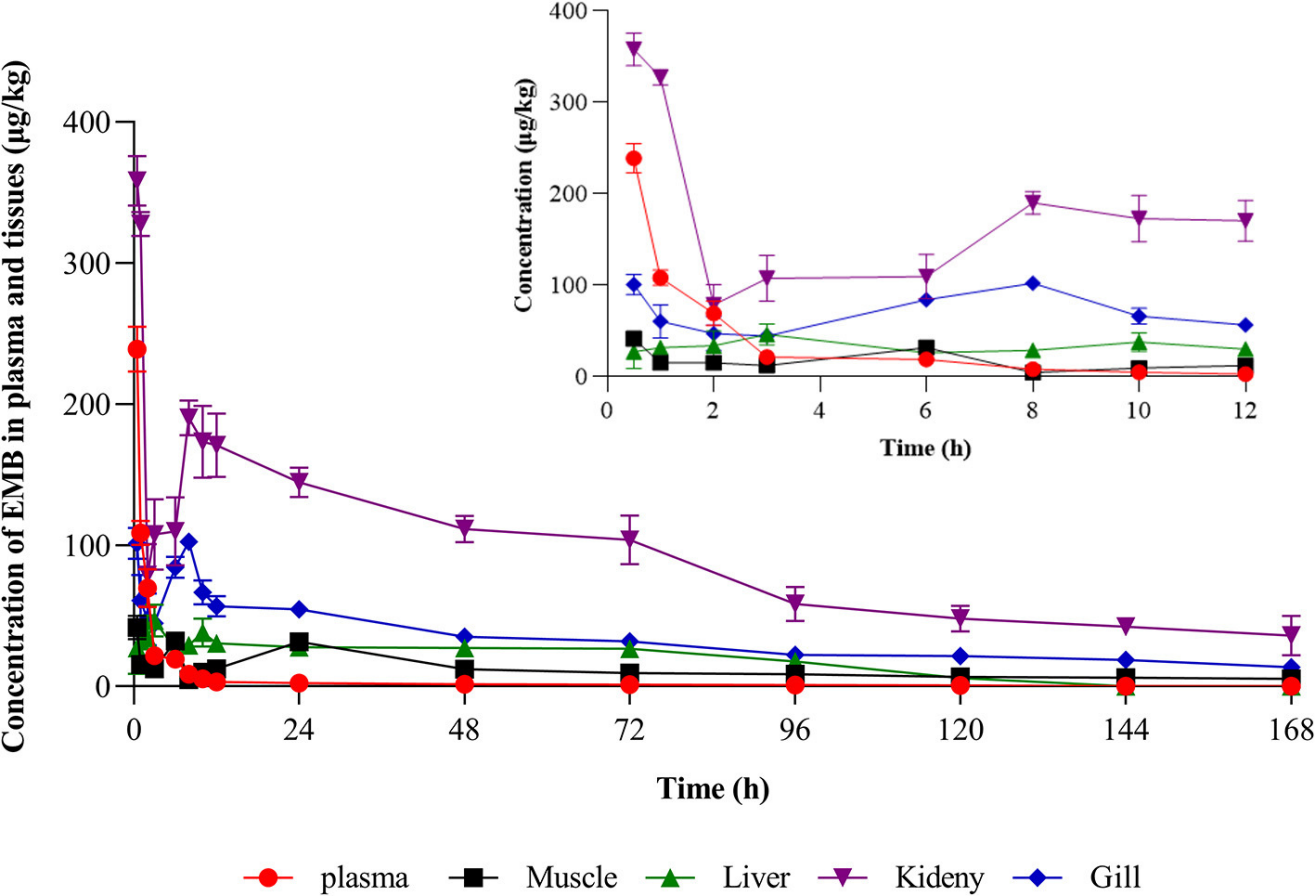
Concentration-time profiles of EMB in tissues of crucian carp after single IV treatment with 50 μg/kg bw at 22 ± 2°C (*n* = 5).

Concentration-time profiles of EMB in muscle plus skin, liver, kidney, and gill after PO treatment with 50, 200, and 500 μg/kg bw and IV treatment with 50 μg/kg bw were shown in Figures 3, 4. Pharmacokinetic parameters of EMB in crucian carp tissues were listed in Tables 4–7.

**Table 4:** Important pharmacokinetic parameters in muscle plus skin of crucian carp following administration with different doses of EMB.

**Parameters**	**Unit**	**Administration**			
		**PO (**μ**g/kg bw)**		**IV (**μ**g/kg bw)**	
		**50**	**200**	**500**	**50**
*λz*	1/h	0.023	0.012	0.008	0.007
*T_1/2*z*_*	h	29.75	56.78	91.62	103.40
*T_*max*_*	h	8.0	8.0	8.0	0.5
*C_*max*_*	μg/kg	71.03	93.95	226.78	41.71
*AUC_0−168_*	h·μg/kg	5,698.62	11,367.16	22,258.31	1,923.50
*MRT_*last*_*	h	66.27	81.02	71.77	60.64

λ*z*, elimination rate constant; *T*_1/2*z*_, terminal elimination half-life; *T*_*max*_, time to attained maximal concentration; *C*_*max*_, observed maximal concentration; *AUC*_0−168_, area under the concentration-time curve from 0 to 168 h; *MRTlast*, mean flow time of drug in body from 0 to 168 h; PO, oral administration; IV, intravenous administration.

**Table 5:** Important pharmacokinetic parameters in liver of crucian carp following administration with different doses of EMB.

**Parameters**	**Unit**	**Administration**			
		**PO (**μ**g/kg bw)**		**IV (**μ**g/kg bw)**	
		**50**	**200**	**500**	**50**
*λz*	1/h	0.031	0.009	0.010	0.031
*T_1/2*z*_*	h	22.51	74.93	64.13	22.22
*T_*max*_*	h	8.0	8.0	8.0	4.0
*C_*max*_*	μg/kg	54.69	199.81	365.10	46.59
*AUC_0−168_*	h·μg/kg	2,870.68	13,342.86	33,285.94	2,871.20
*MRT_*last*_*	h	69.84	62.61	70.41	49.94

λ*z*, elimination rate constant; *T*_1/2*z*_, terminal elimination half-life; *T*_*max*_, time to attained maximal concentration; *C*_*max*_, observed maximal concentration; *AUC*_0−168_, area under the concentration-time curve from 0 to 168 h; *MRTlast*, mean flow time of drug in body from 0 to 168 h; PO, oral administration; IV, intravenous administration.

**Table 6:** Important pharmacokinetic parameters in kidney of crucian carp following administration with different doses of EMB.

**Parameters**	**Unit**	**Administration**			
		**PO (**μ**g/kg bw)**		**IV (**μ**g/kg bw)**	
		**50**	**200**	**500**	**50**
*λz*	1/h	0.006	0.010	0.011	0.006
*T_1/2*z*_*	h	120.77	67.90	63.29	116.93
*T_*max*_*	h	4.0	4.0	4.0	0.5
*C_*max*_*	μg/kg	99.16	194.13	375.91	358.22
*AUC_0−168_*	h·μg/kg	8,042.34	15,709.71	30,902.62	14,675.57
*MRT_*last*_*	h	75.03	59.93	60.79	60.34

λ*z*, elimination rate constant; *T*_1/2*z*_, terminal elimination half-life; *T*_*max*_, time to attained maximal concentration; *C*_*max*_, observed maximal concentration; *AUC*_0−168_, area under the concentration-time curve from 0 to 168 h; *MRTlast*, mean flow time of drug in body from zero to 168 h; PO, oral administration; IV, intravenous administration.

**Table 7:** Important pharmacokinetic parameters in gill of crucian carp following administration with different doses of EMB.

**Parameters**	**Unit**	**Administration**			
		**PO (**μ**g/kg bw)**		**IV (**μ**g/kg bw)**	
		**50**	**200**	**500**	**50**
*λz*	1/h	0.008	0.007	0.004	0.009
*T_1/2*z*_*	h	84.62	100.44	182.21	78.13
*T_*max*_*	h	8.0	8.0	8.0	8.0
*C_*max*_*	μg/kg	104.38	250.13	506.71	102.54
*AUC_0−168_*	h·μg/kg	9,546.20	23,888.25	61,236.51	5,416.48
*MRT_*last*_*	h	70.03	70.78	76.49	61.66

λ*z*, elimination rate constant; *T*_1/2*z*_, terminal elimination half-life; *T*_*max*_, time to attained maximal concentration; *C*_*max*_, observed maximal concentration; *AUC*_0−168_, area under the concentration-time curve from 0 to 168 h; *MRTlast*, mean flow time of drug in body from 0 to 168 h; PO, oral administration; IV, intravenous administration.

Seen from the tissues concentration data, following PO treatment, *C*_*max*_ in various tissues after PO treatment with 50, 200, and 500 μg/kg bw were found to be 71.03, 93.95, and 226.78 μg/kg at 8 h (skinny muscle), 54.69 and 199.81 and 365.10 μg/kg at 8 h (liver), 99.16, 194.13, and 375.91 μg/kg at 4 h (kidney) and 104.38, 250.13, and 506.71 μg/kg at 8 h (gill), respectively. Moreover, *C*_*max*_ in various tissues of crucian carp after IV treatment were found to be 41.71 μg/kg at 0.5 h (skinny muscle), 46.59 μg/kg at 4 h (liver), 358.22 μg/kg at 0.5 h (kidney), and 102.54 μg/kg at 8 h (gill), respectively.

For PO route, the parameters were described. Values of *AUC*_0−168_ for crucian carp after administration with 50, 200, and 500 μg/kg bw were 5,698.62, 11,367.16, and 22,258.31 h·μg/L in skinny muscle, 2,870.68, 13,342.86, and 33,285.94 h·μg/L in liver, 8,042.34, 15,709.71 and 30,902.62 h·μg/L in kidney and 9,546.20, 23,888.25 and 61,236.51 h·μg/L in gill. Meanwhile, *MRT*_*last*_ were 66.27, 81.02, and 71.77 h in skinny muscle, 69.84, 62.61, and 70.41 h in liver, 75.03, 59.93, and 60.79 h in kidney, and 70.03, 70.78, and 76.49 h in gill, respectively. For IV treatment with 50 μg/kg bw, in skinny muscle, liver, kidney, and gill, values of λ*z* were calculated to be 0.007, 0.031, 0.006, and 0.009 1/h, respectively, meanwhile, *T*_1/2*z*_ were found to be 103.40, 22.22, 116.93, and 78.13 h. *MRT*_*last*_ were 60.64, 49.94, 60.34, and 61.66 h and *AUC*_0−168_ were estimated to be 1,923.50, 2,871.20, 14,675.57, and 5,416.48 h·μg/L.

### 3.3. Bioavailability

According to the AUC values and doses of EMB used in crucian carp plasma following PO and IV routes of administration, the absolute oral bioavailability of EMB (F%) in crucian carp was calculated to be 52.70% at doses of 50 μg/kg bw.

### 3.4. Serum biochemical determination

**Figure 5:**
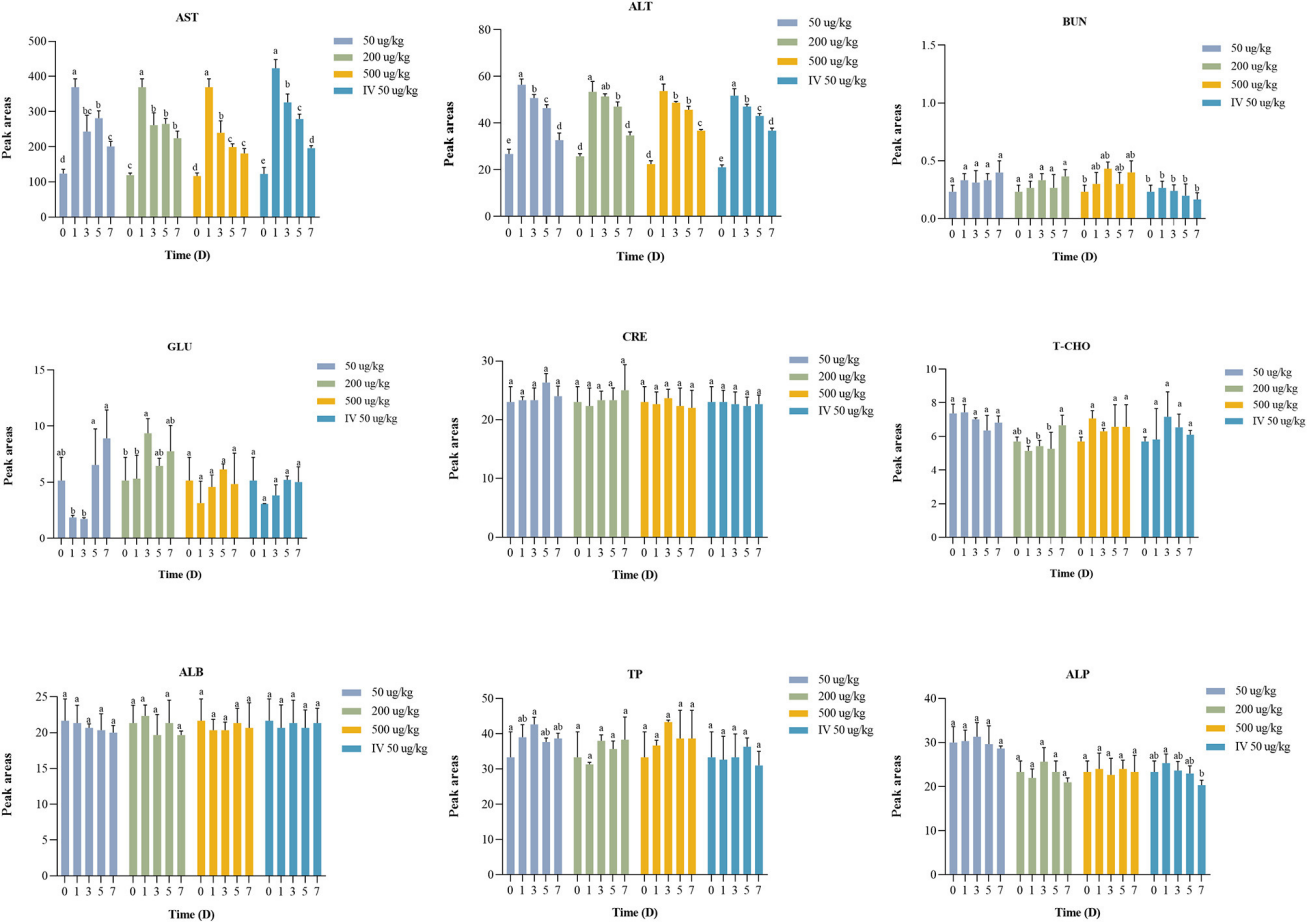
Statistic analysis of serum biochemical indicators in crucian carp exposed to EMB. Significant differences are indicated with different superscript letters (*P* < 0.05).

The serum biochemical analysis results are shown in Figure 5.

The serum biochemical indexes including TP, T-CHO, ALB, ALP, BUN, CRE, and GLU were not significantly different (*P* > 0.05) or regular between the administered group and the control group. After EMB treatment, AST and ALT showed significant differences (*P* < 0.05) at days 1, 3, 5, and 7 compared to the control group (day 0).

## 4. Discussion

### 4.1. Pharmacokinetic patterns and tissue distribution characterization

Our study revealed that in single oral doses at 50, 200, and 500 μg/kg bw for crucian carp, concentrations of EMB in plasma constantly raised while the concentration of administration increased. Meanwhile, concentrations ranged from 4.41 to 53.86 μg/L and the maximum concentration was reached at 8 h after drug administration. This phenomenon indicated that EMB was absorbed quickly, and reached the highest concentration in a short time after completion of administration. Previous studies indicated that fed with EMB 50 μg/kg bw for 7 days, concentrations of EMB in the plasma of salmon were ranging from 6 to 440 μg/L (38) and reached maximum levels of 128 μg/kg on the last day of treatment (day 7) (20). The difference in this concentration change is due to the difference in the number of administrations.

Regarding the plasma pharmacokinetic parameters, our study found that *AUC*_0−168_, *T*_1/2*z*_, and *MRT*_*last*_were found to be dose-dependent, rising with increased dose following PO treatment. *AUC* represents the extent of drug absorption and determines the degree of bioavailability, larger *AUC* values indicate a slower metabolism (39). By comparing two administration routes, *AUC*_0−168_ of EMB in crucian carp plasma by IV route were greater than that following PO treatment with 50 μg/kg bw. Our experimental results showed that the *T*_1/2*z*_ for PO varied from 14.99 to 64.87 h in plasma following administration of 50, 200, and 500 μg/kg bw, respectively. The result also demonstrated that the value of *T*_1/2*z*_ for PO treatment was shorter than that following IV treatment at the same dose. Meanwhile, *MRT*_*last*_varied from 51.88 to 78 h in plasma following PO administration, while it was only 14.71 h following IV administration. These results indicated that the metabolism of EMB is slow, and the increased administration concentration may affect the normal function of the liver and kidney of crucian carp. Furthermore, *T*_1/2*z*_in the plasma of crucian carp in this study was shorter than *T*_1/2*z*_in the plasma of Atlantic salmon, which was determined to be 10.0 days. Combined with the *MRT*_*last*_, the conclusion indicated that EMB remains in crucian carp plasma for a long time, which can play a better treatment effect. Such a conclusion is also recognized in other literature. For example, a conclusion about the validity of EMB for salmon longer than 21 days was presented (14), and similar conclusions have emerged in other studies (13, 40, 41).

The absorption of EMB following a single oral administration was quick, as the maximum concentration in the tissues was detected after the medicated completed 4–8 h, the EMB levels in tissues of crucian carp were liver > gill > kidney > muscle plus skin > plasma. However, when the concentration of EMB was detected 7 days after treatment, the content in gill is highest. We observed a higher level of EMB in gill than in skinny muscle sample likely because the mucous is known to concentrate EMB (20). And EMB can be distributed to many tissues throughout the body, initially easy to be concentrated in the liver, kidney, fat, and muscle tissues (42). After oral administration of different concentrations of EMB, values of *AUC*_0−168_in muscle plus skin, liver, kidney, and gill were increased with increasing administered concentrations. Subsequently, the largest λ*z* was found in liver by two routes of administration. *MRT*_*last*_ in muscle and gill was longer than that in liver and kidney, moreover, at 50 μg/kg bw concentration, values of *MRT*_*last*_in tissues by PO treatment were longer than IV route. In addition, in muscle plus skin and gill, the values of *T*_1/2*z*_ increased with increasing administered concentration. The calculated *T*_1/2*z*_ of EMB in the skinny muscle after IV treatment was 103.40 h as well as after PO treatment were 29.75, 56.78, and 91.62 h, respectively. The result is far less than the studies with 9.2 days in the study of Sevatdal et al. (20), and in another pharmacokinetic study, the *T*_1/2*z*_ in muscle was calculated as 11.1 days, and a clear linear relationship between dose and residual concentration was observed (43).

The study demonstrated that when used in the range of 50–500 μg/kg bw, the metabolism of EMB is relatively slow while providing an important therapeutic effect.

### 4.2. Bioavailability

Bioavailability was proposed by Oser in 1945, referring to the degree and rate of the absorption of the drug (44). Drug content is not the only criterion for determining the curative effect. In addition to drug content, bioavailability should be considered. The bioavailability of EMB was only 38% in cod by determined concentrations of EMB in plasma collected from treated fish after IV (50 μg/kg bw) and PO (50 μg/kg bw) (24). However, the bioavailability of EMB in crucian carp was 52.70%. It showed that EMB has a high bioavailability in crucian carp, and the crucial factor that causes bioavailability to vary is the different fish species.

### 4.3. Serum biochemical indices analysis

The serum biochemical indexes including TP, T-CHO, ALB, ALP, BUN, CRE, and GLU were not significantly different (*P* > 0.05) from the control group. In contrast, AST and ALT in experimental groups exhibited significant differences (*P* < 0.05) compared with the control group. In summary, the results of our study indicated that EMB would induce altered serum parameters.

AST and ALT are important indicator enzymes of the liver (45). The contents of AST and ALT in tilapia were related to hepatic injury (46). In comparison with the control group, ALT and AST levels were increased after administration. In brief, the results of our study indicated that EMB would induce serum parameters and EMB has an effect on liver when metabolized in crucian carp. However, the single PO treatment showed a significant increase in AST and ALT levels on day 1 (*P* < 0.05), with the content gradually decreasing after day 1, and it still showed a downward trend until day 7, which was still significantly higher than those of the control group.

The effect of EMB on the biochemical parameters of rainbow trout was studied. It was found that the fish was administered with EMB of 50 μg/kg bw daily for 21 consecutive days, and the result showed that AST content was increased (22). Increased ALT and AST levels obviously projected EMB as an inducer of hepatic dysfunction in Nile tilapia possibly due to damage of hepatic cells, disrupted Kreb's cycle, and other hepato-cellular damage (46, 47).

The levels of ALT and AST content proved that in the range of 50–500 μg/kg bw EMB stimulated the crucian carp, but the content became normal over time, indicating that there was no substantial damage caused to the body, meanwhile, it was also proved in previous studies (12, 19, 48).

The results of the present study suggested that the effects of EMB on serum biochemical indices are revocable. Nevertheless, it did not reach normalcy after 7 days of dosing. Those data would provide a valid reference for researchers and aquaculturists in assessing the health condition of cultured crucian carp after EMB administration in tropical conditions.

## 5. Conclusion

This study revealed the regularities of pharmacokinetics, bioavailability, and serum biochemical indices of EMB in crucian carp following two administration routes of multiple dose levels. EMB has the characteristics of quick absorption and slow elimination in crucian carp, and accumulates easily in gill and skinny muscle. In addition, with a high bioavailability by PO route in crucian carp, EMB can achieve a better therapeutic effect in practical application, and EMB has a large safe concentration range.

## Data availability statement

The original contributions presented in the study are included in the article/supplementary material, further inquiries can be directed to the corresponding author.

## Ethics statement

The animal study was reviewed and approved by the Fish Ethics Committee of Yangtze River Fisheries Research Institute, Chinese Academy of Fishery Sciences, Wuhan, China (ID 2021-Liu Yongtao-03).

## Author contributions

RS: conceptualization, methodology, investigation, data curation, and writing—original draft. YL: resources, writing—review and editing, and project administration. XA: supervision and resources. XD and XZ: software and validation. All authors contributed to the article and approved the submitted version.
